# Identification of a Novel Specific Cucurbitadienol Synthase Allele in *Siraitia grosvenorii* Correlates with High Catalytic Efficiency

**DOI:** 10.3390/molecules24030627

**Published:** 2019-02-11

**Authors:** Jing Qiao, Zuliang Luo, Zhe Gu, Yanling Zhang, Xindan Zhang, Xiaojun Ma

**Affiliations:** 1 Institute of Medicinal Plant Development, Chinese Academy of Medical Sciences & Peking Union Medical College, Beijing 100193, China; qiaojing_happy@126.com (J.Q.); zuliangluo@163.com (Z.L.); gudatuier@163.com (Z.G.); 2 Guilin GFS Monk Fruit Corp., Guilin 541006, China; zhangyanling2008y@163.com (Y.Z.); zhangxindan2008y@163.com (X.Z.)

**Keywords:** *Siraitia grosvenorii*, molecular breeding, site-directed mutant, mogrosides, cucurbitadienol synthase, single nucleotide polymorphism (SNP)

## Abstract

Mogrosides, the main bioactive compounds isolated from the fruits of *Siraitia grosvenorii*, are a group of cucurbitane-type triterpenoid glycosides that exhibit a wide range of notable biological activities and are commercially available worldwide as natural sweeteners. However, the extraction cost is high due to their relatively low contents in plants. Therefore, molecular breeding needs to be achieved when conventional plant breeding can hardly improve the quality so far. In this study, the levels of 21 active mogrosides and two precursors in 15 *S. grosvenorii* varieties were determined by HPLC-MS/MS and GC-MS, respectively. The results showed that the variations in mogroside V content may be caused by the accumulation of cucurbitadienol. Furthermore, a total of four wild-type cucurbitadienol synthase protein variants (50R573L, 50C573L, 50R573Q, and 50C573Q) based on two missense mutation single nucleotide polymorphism (SNP) sites were discovered. An in vitro enzyme reaction analysis indicated that 50R573L had the highest activity, with a specific activity of 10.24 nmol min^−1^ mg^−1^. In addition, a site-directed mutant, namely, 50K573L, showed a 33% enhancement of catalytic efficiency compared to wild-type 50R573L. Our findings identify a novel cucurbitadienol synthase allele correlates with high catalytic efficiency. These results are valuable for the molecular breeding of luohanguo.

## 1. Introduction

*Siraitia grosvenorii* (luohanguo or monk fruit) is an herbaceous perennial of the Cucurbitaceae family. It is principally cultivated in Guilin city, Guangxi Province, China [1]. The fruit of *S. grosvenorii* has been used in China as a natural sweetener and as a folk remedy for the treatment of lung congestion, sore throat and constipation for hundreds of years [2]. To date, luohanguo products have been approved as dietary supplements in Japan, the US, New Zealand and Australia [3,4]. Mogrosides, the major bioactive components isolated from the fruits of *S. grosvenorii*, are a mixture of cucurbitane-type triterpenoid glycosides that have been proven to be powerful and zero-calorie sweeteners and can hence be used as a sucrose substitute for patients with diabetes and patients who are obese [5].

Because of their complex structures (Table A1), the chemical synthesis of these compounds is inherently difficult [6]. Currently, these valuable chemicals are mainly produced through their extraction from the fruits of *S. grosvenorii*. With the rapid rise in market demand, the production of luohanguo extracts has increased rapidly from two tons in 2002 to 60 tons in 2007, becoming one of the fastest growing industries of traditional Chinese medicine extracts [7]. However, the extraction yield of these ingredients is limited by difficulties in *S. grosvenorii* cultivation, including a requirement for heavy artificial pollination, a scarcity of appropriate cultivatable land and a high purification cost due to the presence of seeds [8,9]. In addition, the low contents of the main active components also result in a high cost of extraction. According to the statistics of our team, the extraction cost can be reduced by 1% when the mogroside V (MV) content increases by 0.1%. Therefore, the production of high-quality *S. grosvenorii* is an urgent issue for research and in the production field.

In recent years, our team has selected many new *S. grosvenorii* varieties with better agronomic traits, such as higher yield, improved resistance to various diseases and seedless fruits [10,11,12,13]. In this study, we chose 15 *S. grosvenorii* varieties (E1, E2, E3, E5, E12, E23, E29, S2, S3, S10, S13, C2, C3, C6 and W4). From these varieties, elite germplasms, which constitute an important resource for *S. grosvenorii* breeding, may be developed. The macroscopic characteristics of the 15 *S. grosvenorii* varieties are very similar, especially beyond the flowering and fruiting periods. However, the quality varies dramatically. Therefore, further evaluations of *S. grosvenorii* germplasms based on the active component content are important to better serve molecular breeding purposes. Because all of these varieties are cultivated under the same conditions, we hypothesize that effective genetic polymorphisms in functional genes involved in MV biosynthesis are one of the most important reasons for the MV content change.

**Figure 1:**
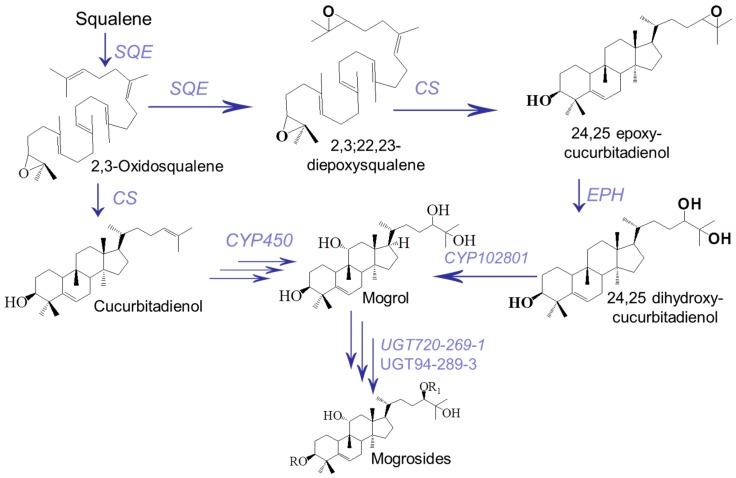
Genes involved in the mogroside biosynthesis pathway. 2,3-oxidosqualene is converted cucurbitadienol by cucurbitadienol synthase (*CS*), while cucurbitadienol is further converted to mogrosides through epoxide hydrolase (*EPH*), cytochrome P450s (*CYP450*) and UDP-glucosyltransferases (*UGT*). Single arrows represent the one-step conversions, while triple arrows represent multiple steps.

To date, key genes involved in the biosynthesis of mogrosides have been successfully cloned and characterized, including genes from five enzyme families: the squalene epoxidase (SQE) [14], cucurbitadienol synthase (CS) [15], epoxide hydrolase (EPH), cytochrome P450 (CYP450) [2,9] and UDP-glucosyltransferase (UGT) families (Figure 1) [16]. Squalene is thought to be the initial substrate and precursor for triterpenoid and sterol biosynthesis. SQE has been generally recognized as the common rate-limiting enzyme in the common pathway from mevalonate (MVA) and methylerythritol phosphate (MEP) pathways, catalyzing squalene to 2,3-oxidosqualene [17,18]. In *S. grosvenorii*, the initial step in the biosynthesis of cucurbitane-type mogrosides is the cyclization of 2,3-oxidosqualene to form the triterpenoid skeleton of cucurbitadienol, which is catalyzed by CS. EPH and CYP450 further oxidize cucurbitadienols to produce mogrol, which is glycosylated by UGT to form MV.

Single nucleotide polymorphism (SNP) derived markers, identified in coding sequences of different genes, have been developed to discriminate very similar cultivars [19,20,21]. Technological improvements make the use of SNP attractive for high throughput use in marker-assisted breeding, for population studies [22] and to develop high-density linkage maps for map-based gene discovery [23,24]. In the resulting 2.44 Mbp of aligned sequence of soybean, a total of 5551 SNPs were discovered, including 4712 single-base changes and 839 indels for an average nucleotide diversity of Theta = 0.000997 [25]. No SNP were analyzed in *S grosvenorii* so far though the genome was assembled successfully [26].

This study analyzes the variation of 23 profiles in 15 *S. grosvenorii* varieties aimed to identify favorite allele of SgSC gene that can be used increase the production of them in the breeding program. A holistic targeted secondary metabolomics analysis was conducted to quantitatively determine the contents of 21 mogrosides and two intermediates in 15 varieties of luohanguo samples using high-performance liquid chromatography with tandem mass spectrometry (HPLC-MS/MS) or gas chromatography mass spectrometry (GC-MS). *SgCS* genes from a total of 15 *S. grosvenorii* varieties were cloned to investigate the enzyme activity of the allele gene products. Using a combined approach that relied on SNP analyses, yeast expression, and site-directed mutagenesis, the key amino acid residues underlying triterpene product efficiency were identified, providing new insight into the molecular breeding of high-quality luohanguo.

## 2. Results

### 2.1. Determination of the Levels of 21 Mogrosides in 15 S. grosvenorii Varieties

**Figure 2:**
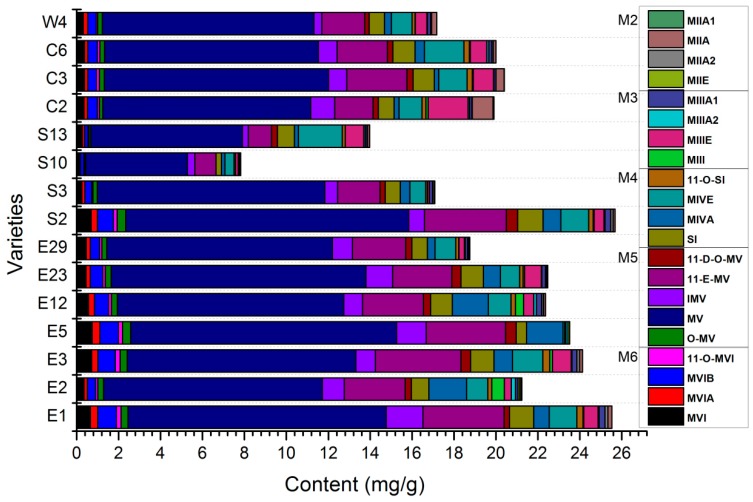
Graphical representation of the content of each mogroside and the total content in the different varieties of luohanguo. MV was the predominant component in all samples. 11-E-MV and MIVE were the second and third most abundant sweet mogrosides in the tested samples, respectively. S2 and E1 can be used as the elite germ to breeders for *S. grosvenorii* quality improvement. Abbreviations: MIIA1, mogroside IIA1; MIIA, mogroside IIA; MIIA2, mogroside IIA2; MIIE, mogroside IIE; MIIIA1, mogroside IIIA1; MIIIA2, mogroside IIIA2; MIIIE, mogroside IIIE; MIII, mogroside III; 11-O-SI, 11-O-siamenoside I; MIVE, mogroside IVE; MIVA, mogroside IVA; SI, siamenoside I; 11-D-O-MV, 11-deoxymogroside V; 11-E-MV, 11-epi-mogroside V; IMV, isomogroside V; MV, mogroside V; O-MV, 11-oxo-mogroside V; 11-O-MVI, 11-oxo-mogroside VI; MVIB, mogroside VIB; MVIA, mogroside VI A; MVI, mogroside VI.

The developed HPLC-MS/MS method was applied to determine the levels of the 21 mogrosides in the fruits of different *S. grosvenorii* varieties. The quantitative analyses were performed by means of an external standard method [27]. The results are summarized in Table A2, and a graphical representation of the results is shown in Figure 2. The content of each targeted mogroside and the total content of each targeted analyte in the fruits of different *S. grosvenorii* varieties varied dramatically (7.77–19.97 mg/g). Among these, the level of M5 was much higher than that of any other mogroside. In the fruit of *S. grosvenorii*, the average total content of M5 was 63.86-fold, 15.86-fold, 5.05-fold and 11.26-fold higher than the contents of M2, M3, M4 and M6, respectively. According to a previous report, ripe fruits mainly contain M5, while unripe fruits have higher levels of M2 and M3. The M5 content significantly increased, while the levels of M2 and M3 dramatically decreased with increasing growing time (disappeared after 70 DAA) [28,29]. In our study, we did not find MIIA1 in most of the samples and found only a small amount of MIII. The average values of MIII, MIIIE, MIIIA1, and MIIIA2 were 0.10, 0.62, 0.06 and 0.15 mg/g, respectively.

In terms of individual constituents, MV was the predominant component in all samples. The MV content accounts for 45.23–63.58% of the total content of the 21 targeted analytes. This result is consistent with previous reports that MV was found in a sample of whole fruits of *S. grosvenorii* at levels of 49.29–66.96% [27]. Moreover, its content was in the range of 4.86–13.49 mg/g in 15 varieties of luohanguo samples, all of whose contents, except the S10 variety, were all higher than the 5 mg/g set in the Chinese Pharmacopeia. Then, 11-E-MV and MIVE were the second and third most abundant sweet mogrosides in the tested samples, respectively. The average values of 11-E-MV and MIVE were 2.67 and 1.12 mg/g, respectively.

MIIE, MIIIX, MIVA and SI are the biosynthetic precursor components for the synthesis of MV. MIIE was detected in E2, E3, E5 and E12 but not in the other samples. MIIIX was not detected because the standard was unavailable. The MIVA content ranged from 0.16 to 1.80 mg/g, with a mean value of 0.73 mg/g. As the sweetest among the mogrosides, SI was the fifth most abundant sweet mogroside in the tested varieties. The mean content of SI was 0.81 mg/g and ranged from 0.26 to 1.21 mg/g. The results showed that the contents of the mogrosides were significantly different between the different varieties of *S. grosvenorii*.

**Figure A1:**
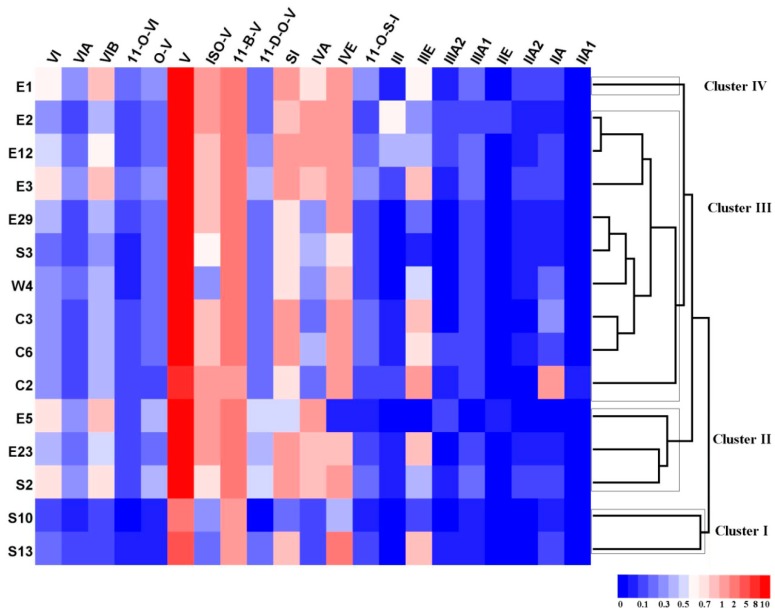
Hierarchical clustering analysis of *S. grosvenorii* varieties. Species were grouped into four main clusters. Species S10 and S13 were categorized into Cluster I. S2, E23 and E5 were grouped into Cluster II. E1 was defined into third distinct cluster (Cluster III). The remaining species including C2, C3, C6, W4, S3, E29, E3, E12 and E2 were put into Cluster IV.

In order to explore the clustering of *S. grosvenorii* samples from different species, hierarchical clustering analysis (HCA) was performed on 21 mogrosides content. 15 species were analyzed using HCA of the peak areas of the 21 analytical markers in the LC–MS/MS. The between-groups linkage method was chosen as average linkage and the squared Euclidean distance was selected to establish clusters, and to assess the resemblance of the 15 species of samples. The resulting hierarchical cluster heat-map in Figure A1 produced well-defined clusters and showed the peak area profile of the 21 mogrosides peaks. Species were obviously grouped into four main clusters. Species S10 and S13 were categorized into Cluster I. S2, E23 and E5 were grouped into Cluster II. E1 was defined into third distinct cluster (Cluster III). The remaining species including C2, C3, C6, W4, S3, E29, E3, E12 and E2 were put into Cluster IV. These results agreed with a simple visual comparison of content analysis, and with the ratio among different mogrosides (Figure 2), which can reflect their quality. Combining these findings, we further predicted that the magnitude of the four groups’ quality should be ordered as follows: Cluster III > Cluster II > Cluster IV > Cluster I. Although the total mogrosides content was not different, significant variation (*p* < 0.05) in MV concentration was observed for the elite germplasm lines S2 and E1. These results showed that species S2 and E1 could be useful to breeders for *S. grosvenorii* quality improvement.

### 2.2. The Determination of Squalene and Cucurbitadienol Levels in the Fruit of S. grosvenorii

**Figure 3:**
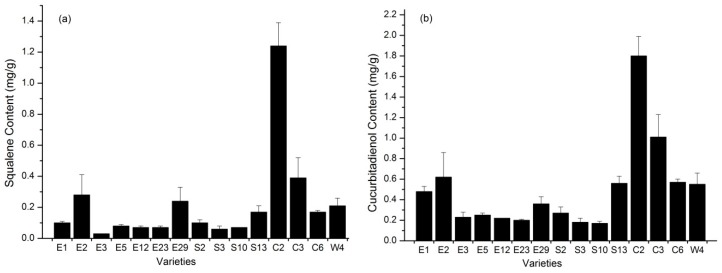
Graphical representation of the (**a**) squalene and (**b**) cucurbitadienol content in the different varieties of luohanguo.

When the contents of related intermediates (squalene, cucurbitadienol) in luohanguo were analyzed by GC-MS, the results showed significant variations among the different varieties (*p* < 0.05 and 0.01, respectively). For example, the squalene content of C2 was 1.24 mg/g, whereas the squalene content of E3 was only 0.03 mg/g (Figure 3a). Similar to squalene, the contents of cucurbitadienol in the different varieties of luohanguo were obviously different. The mean content of cucurbitadienol was 0.50 mg/g and ranged from 0.17 to 1.80 mg/g (Figure 3b).

The results revealed a wide range of variability among the 15 *S. grosvenorii* varieties for 23 quantitative components (Appendix Table A2 and Table A3). The squalene and cucurbitadienol coefficients of variation (CVs) were higher than the mogroside CV. The CVs of squalene and cucurbitadienol were 135.03 and 86.03%, respectively. In contrast, the lowest CV belonged to the MV (21.12%). Because the M2, M3 and M4 contents were very low in ripe fruit, we speculated that catalysis by UGTs is not a rate-limiting step for MV biosynthesis in plants. Moreover, squalene and cucurbitadienol, the precursor of mogrosides, accumulates in fresh fruit. SgCS and CYP450 are the enzymes that can catalyze these two substrates, respectively. We proposed that the conversion of squalene and cucurbitadienol through improved activity of SgCS and CYP450 was proportional to the accumulation of MV. In this paper, we mainly focus on the enzyme catalytic efficiency of SgCS, and we think it is necessary to study the catalytic efficiency of CYP450 in the future. When cucurbitadienol increases under high SgCS catalytic efficiency, the content of M2, M3 and M4 may increase, and then it may lead to the increase of pharmaceutical ingredients MV via the same biosynthetic route they shared.

### 2.3. SNP Identification in ORF Region of the SgCS Gene

**Figure 4:**
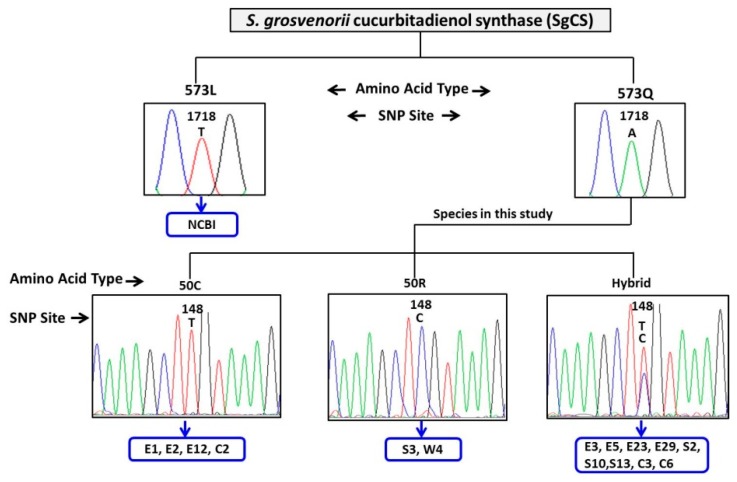
Missense mutations of SgCS in the 15 *S. grosvenorii* varieties. Four types of SgCS protein variants (50R573L, 50C573L, 50R573Q and 50C573Q) based on the missense mutation SNP sites were identified. The number is the position of the protein sequence, R, C, L, and Q is corresponding amino acid Arg, Cys, Leu, Gln.

**Figure A2:**
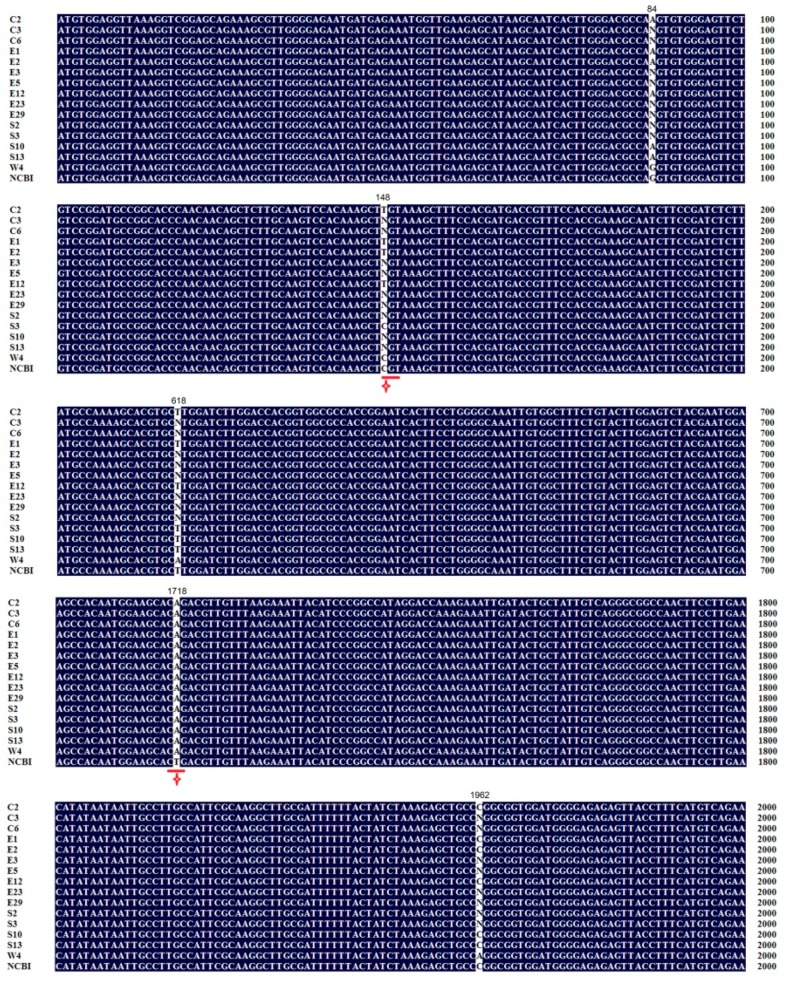
Alignment of PCR fragment showing the SNP sites of SgCS. Missense mutation are marked with stars.

SgCS is a member of the oxidosqualene cyclase (*OSC*) gene family and catalyzes the cyclization of 2,3-oxidosqualene to cucurbitadienol. This step, catalyzed by OSCs, is the key branch-point leading to triterpenoid or sterol synthesis [30,31,32]. A 2800 bp full-length cDNA sequence of the *SgCS* gene encoding a 759-residue protein (between 200 and 2479 bp) was obtained from 15 varieties. To detect sequence polymorphisms among different cultivars, we aligned the ORF sequences, which revealed a total of 4 SNPs among 15 varieties. No InDels were distributed in the ORF region of the *SgCS* gene. The changes in nucleotides at the 84 and 148 sites were due to transitions (A-G or C-T), whereas transversions (A-T, A-C) existed in 618 and 1962 sites. Of the SNPs, only 148 sites were missense mutations that caused changes in amino acid sequences between Arg (R) and Cys (C). To date, a universal platform that can allow data comparisons across different laboratories has been used. The sequence alignment analysis of the 15 SgCS clones against the expressed sequence tags (ESTs) within GenBank (GenBank accession number: HQ128567) identified another SNP site in 1718 bp, and this SNP is a missense mutation (Figure A2). Therefore, four types of SgCS protein variants (50R573L, 50C573L, 50R573Q and 50C573Q) based on the missense mutation SNP sites (Figure 4) were identified.

### 2.4. Activity Comparison

**Figure 5:**
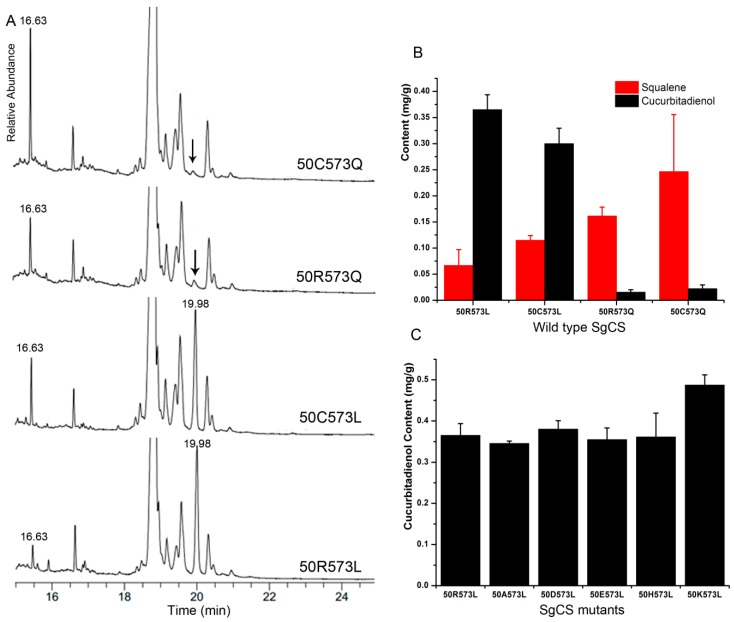
Production of cucurbitadienol by an engineered *S. cerevisiae* strain. (**A**) GC-MS analysis of cell extracts of BY4742, which had wild-type SgCS. Squalene (RT = 16.63 min) and cucurbitadienol (RT = 19.98 min) (**B**) Squalene and cucurbitadienol production by the different yeast strains with four wild-type SgCSs (50R573L, 50C573L, 50R573Q and 50C573Q). (**C**) Cucurbitadienol production by the different yeast strains with SgCS mutants (50A573L, 50D573L, 50E573L, 50H573L and 50K573L).

To measure cucurbitadienol synthetic activity, the four different types (50R573L, 50C573L, 50R573Q and 50C573Q) of *CS* genes from *S. grosvenorii* were codon-optimized, synthesized and subcloned into the yeast expression vector pCEV-G4-Km. The resulting recombinant plasmids and the empty vector pCEV-G4-Km, used as a negative control, were transformed into the BY4742 strain. Colonies were randomly picked from the plate and verified by PCR amplification. All correct colonies were cultivated in yeast extract peptone dextrose medium (YPD) medium with 2% glucose for 3 days. GC-MS analysis of the cell extract confirmed production of cucurbitadienol. Protein variants 50R573L and 50C573L generated products with similar cucurbitadienol yields (0.365 and 0.300 mg/g yeast cells), whereas 50R573Q and 50C573Q produced much smaller amounts of cucurbitadienol (0.015 and 0.022 mg/g yeast cells). At the same time, 50C573Q accumulated 3.7 times more squalene than 50R573L. These results demonstrate that SgCS variants from cultivated *S. grosvenorii* are highly divergent, as inferred from their product contents (Figure 5a,b).

The approach adopted here was to search for the SNP site of the gene that is known to be closely linked to a trait of interest, namely, content. Unexpectedly, SNPs were tested for significant deviation from equal distribution between the two groups of high cucurbitadienol content and low cucurbitadienol content with the χ^2^ test. The results suggested that there were no significant relationships between these SNP sites and cucurbitadienol content in the different *S. grosvenorii* varieties. A possible explanation is that the accumulation of cucurbitadienol is a dynamic process because these classes of secondary metabolites share a common synthetic pathway [33]. The cucurbitadienol content in plants depends not only on the activity of SgCS but also on the downstream enzyme that can use cucurbitadienol as a substrate for synthase mogrosides.

Measuring Km values in the presence of detergents is notoriously difficult because the concentrations of the substrate and enzyme are distorted by the biphasic aqueous and micellar system [34,35]. Although most of the enzyme and substrate are probably constrained to the restricted volume of the micelle, the soluble proportion is not readily determined. We consequently compared the catalytic competence of these enzymes using homogenate assays with substrate at a concentration (250 µM) well above the literature Km of 25–125 µM described for several plant cyclases. The activities of purified variants were tested, and the results are shown in Table 1. 50R573L showed the highest activity as the best variant. The specific activity of 50C573L showed a slight decline, with a loss of approximately 20% of activity compared to 50R573L. Inferences from in vitro experiments of 50R573L and 50C573L were nearly in agreement with the results of the in vivo experiments, indicating that the in vivo assay can serve as an alternative to the in vitro assay when the enzyme activities of 50R573Q and 50C573Q are too weak for measurements of Km and *k*_cat_.

The amino acid sequence of SgCS shares high similarity with the sequences of CSs (85% with CcCS from *C. colocynthi*s, 89% with CpCS from *C. pepo* and 84% with CsCS from *C. sativus*). Residues 50R and 573L in SgCS correspond to 52R and 578L, respectively, in CcCS and CpCS and 70R and 599L, respectively, in CsCS. Previous study has shown that SgCS, which had the highest cucurbitadienol yield, is the wild-type CS enzyme with highest catalytic efficiency [36]. Therefore, SgCS can be used as a guideline for other CS enzyme modification.

### 2.5. Improving the Activity of SgCS by Site-Directed Mutagenesis

To rationally produce a more catalytically efficient SgCS, site-directed mutagenesis of 50R573L was performed. We constructed five variants (50A573L, 50D573L, 50E573L, 50H573L and 50K573L) to verify the effects of 50 amino acid residues in SgCS. The subsequent enzyme assay by GC-MS revealed that the cucurbitadienol contents of 50A573L, 50E573L and 50H573L were reduced slightly in comparison with that of the wild-type, whereas that of 50K573L increased by approximately 33.5% (Figure 5c). Notably, the catalytic efficiency of the 50K573L mutant was enhanced by approximately 1.62-fold compared to that of 50C573L. These results showed that lysine was the optimal amino acid for the 50 position, with higher activity. Therefore, to rationally produce more catalytically efficient SgCSs, site-saturation mutagenesis of the 50 position needs to be further performed individually.

## 3. Discussion

*S. grosvenorii* is an important herbal crop with multiple economic and pharmacological uses. Mogrosides, the main effective components of luohanguo, are partial substitutes for sucrose because of their extremely sweet and noncaloric characteristics, and increasing progress is being made in terms of molecular breeding and purification processes. That the production and distribution of mogrosides in monk fruits is regulated by genetic factors, growth times, environmental factors, physiological factors, and chemical factors is generally accepted. In this study, 15 varieties of *S. grosvenorii* were cultivated under the same conditions and harvested at the same time, suggesting that the differences in the active component contents were most likely caused by genetic differences. We hypothesize that effective genetic polymorphisms in functional genes involved in MV biosynthesis constitute one of the most important mechanisms of changes in MV content.

The biosynthesis pathway of mogrosides has been extensively studied, and several genes have been identified. The initial committed step in the biosynthesis of cucurbitane-type mogrosides is the cyclization of 2,3-oxidosqualene to form the triterpenoid skeleton of cucurbitadienol. This step is catalyzed by CS, which has been functionally characterized in several plants, including *Cucurbita pepo* [37], *Citrullus colocynthis* [38], *Cucumis sativus* [39] and *S. grosvenorii* [15]. However, no kinetic data for CS from these plants have been reported. In this study, the two key amino acid sites that determine the enzyme activity of cucurbitadienol synthesis were successfully identified through comprehensive SNP analysis among 15 varieties of *S. grosvenorii*. An in vitro study indicated that the wild-type 50R573L enzyme has quite good efficiency considering that many triterpene cyclases have very low efficiencies [34]. The apparent *Km* values for 3(S)-oxisqualene were determined to be 55µM for lanosterol cyclase in rat [40] and 33.8 µM for β-Amyrin cyclase in *E. tirucalli* L. [35], the reported values for pea cyclases being 25 and 50 µM, respectively [41]. The use of SNP information combined with site-directed mutation is an effective approach to enhance enzyme activity. In this study, this approach enabled us to identify a mutant 50K573L that had 133% efficiency compared to wild-type 50R573L. To rationally produce more catalytically efficient SgCSs, site-saturation mutagenesis of the 50 position needs to be further performed individually.

During the evolutionary process, plants have perfected their systems to produce diverse catalogs of compounds in adaptations to both natural and artificial selection processes. Rapid advances in genome sequencing technologies have greatly accelerated studies of the molecular basis underlying these evolutionary events [42,43], which provide deep insights into nature’s strategy for survival in a specific ecological niche. Phylogenetic analysis showed that *S. grosvenorii* diverged from the Cucurbitaceae family approximately 40.95 million years ago [26]. We generated and analyzed a multiple sequence alignment to test whether the 50 and 573 amino sites of CS were conserved in the Cucurbitaceae plant family. The substitution of 573L with 573Q resulted in an almost complete loss of function in terms of producing cucurbitadienol, indicating that this amino acid site may serve as an active-site residue for 2,3-oxidosqualene cyclization to cucurbitadienol. Previous study showed that a point mutations Cys (C) to Tyr (Y) at position 393 of cucumber CS which disables the catalytic capacity of CcCS inhibiting bitterness biosynthesis in cucumber [39]. However, in contrast to the well-studied functionally characterized SgCS, key amino acid residues responsible for the formation of cucurbitadienol have not been identified. Therefore, further studies based on homologous modeling and site-directed mutagenesis should be carried out.

Integrating “good genes”, into high-yielding, high-content cultivars continues to be one of primary objectives of many breeding programs [44]. Although substantial efforts have been made during the past twenty years to develop an optimal yield of the final product MV *S. grosvenorii* cultivars using conventional plant breeding methods, there has been limited success in achieving the desired goal [45]. After crossings one needs to screen thousands of individual plants for their performance, which is time consuming and costly as the plants have to be cultivated till there are fruits. With the advent of molecular biology techniques, it was presumed that developing high-quality cultivars would be convenient and relatively less time consuming. Results from our studies identified genetic sources of high SgCS efficiency that could be useful to breeders for *S. grosvenorii* improvement. 

In *S. grosvenorii*, squalene is converted to cucurbitadienol by *SQE,* and cucurbitadienol is further converted to mogroside compounds by *CYP450* and *UGT*. After the metabolic pathway of mogroside in *S. grosvenorii* was identified [2,9,16,26,46], the SNP sites of *SQE*, *CYP450*s and *UGT* could be discovered to reveal a new hypothesis that effective genetic polymorphisms in functional genes involved in MV biosynthesis can result in changes in MV content. Due to the large collection of high-efficiency mutants, the further step for breeding the “super” *S. grosvenorii* cultivar should focus on cotransformation of all of these homozygous functional enzymes with high activity into the elite germplasm S2 or E1. Additionally, these mutants can be valuable gene resources for the production of mogrosides by metabolic engineering. 

## 4. Materials and Methods 

### 4.1. Chemicals and Reagents

The following reference compounds were purchased from Chengdu Must Bio-Technology Co., Ltd. (Sichuan, China): mogroside IIA (MIIA), mogroside IIA1 (MIIA1), mogroside IIA2 (MIIA2), mogroside IIE (MIIE), mogroside III (MIII), mogroside IIIE (MIIIE), mogroside IIIA1 (MIIIA1), mogroside IIIA2 (MIIIA2), 11-O-siamenoside I (11-O-SI), siamenoside I (SI), mogroside IVA (MIVA), mogroside IVE (MIVE), 11-deoxymogroside V (11-D-O-MV), 11-epi-mogroside V (11-E-MV), MV, 11-oxo-mogroside V (O-MV), isomogroside V (IMV), 11-oxo-mogroside VI (11-O-MVI), mogroside VI (MVI), mogroside VI A (MVIA) and mogroside VI B (MVIB), all with purity > 98% as determined by HPLC-DAD-ELSD. Squalene and 3(S)-oxidosqualene were obtained from J&K Scientific Ltd. (Beijing, China). HPLC-grade formic acid, methanol, acetonitrile and hexane were obtained from Fisher (Emerson, IA, USA). Other reagents and chemicals were of analytical grade and purchased from Sinopharm Chemical Regent Beijing (Beijing, China). Deionized water was prepared using a Milli-Q purification system (Millipore, MA, USA).

Plant genomic DNA kits and DNA Marker II were obtained from TianGen Biotech Co. Ltd. (Beijing, China). KOD-Plus DNA Polymerase was purchased from TOYOBO Biotech Co., Ltd. (Shanghai, China). Primer synthesis and DNA sequencing were conducted at Sangon Biotech Company (Beijing, China). Restriction enzymes and DNA ligase were purchased from Takara Biotechnology Co. Ltd. (Dalian, China). The host strain *S. cerevisiae* BY4742 was purchased from Invitrogen (Carlsbad, CA, USA). The yeast pCEV-G4-Km expression plasmid (Addgene plasmid #46819) was a gift from Lars Nielsen and Claudia Vickers [47]. Other reagents were purchased from Beijing Chemical Corporation (Beijing, China) unless otherwise specified.

### 4.2. Sample Collection and Preparation

Fifteen *S. grosvenorii* varieties were cultivated on the farm of Lingui Monk Fruit Production Base (25.2° N, 110.0° E, Guangxi, China) and harvested 80 days after flowering. The samples were authenticated as the fruits of *S grosvenorii* Swingle by Prof. Xiaojun Ma from the Institute of Medicinal Plant Development. All fruits were sealed in plastic bags and stored at 20 °C before analysis. Plant leaves were cut and frozen in liquid nitrogen and saved at −80 °C for DNA extraction.

The dry fruit samples were powdered to a homogeneous size (ca. 50 mesh) by a disintegrator (Shanghai Shuli Instrument, Shanghai, China). Approximately 0.5 g of powder from each sample was accurately weighed and introduced into a 50-mL capped conical flask with 25 mL of methanol/water (80:20, *v*/*v*). The flask was sealed and sonicated in a KQ-300 ultrasonic water bath (Kunshan Ultrasonic Instrument, Jiangsu, China) operating at 40 kHz with an output power of 300 W for 30 min at room temperature. A duplicate extract was prepared. Both extracts were mixed and transferred into a volumetric flask and then diluted to 100 mL with methanol/water (80:20, *v*/*v*) and filtered through a 0.22 μm microporous membrane.

For the analysis of the nonpolar compounds squalene and cucurbitadienol from *S. grosvenorii*, 50 mL of the above mentioned methanol/water extracts was extracted three times with the same volume of n-hexane. The combined extracts were dried under reduced pressure distillation and dissolved in 1 mL of n-hexane.

### 4.3. LC–MS/MS Analysis of 21 Mogrosides

The HPLC system consisted of an Agilent Technologies 1260 Series LC system (Agilent, USA) equipped with an automatic degasser, a quaternary pump, and an autosampler. Chromatographic separations were performed on an Agilent Poroshell 120 SB C18 column (100 mm × 2.1 mm, 2.7 μm) by gradient elution using a mobile phase consisting of (A) water (containing 0.1% formic acid) and (B) acetonitrile with the following gradient procedure: 0–8 min 25% B, 11 min 80% B, 11.01–11.50 min 80% B and 11.51–15.0 min 20% B, with a flow rate of 0.20 mL/min. The injection volume was 2.0 μL.

The column effluent was monitored using a 4000 QTRAP^®^ LC–MS/MS (AB Sciex, Toronto, Canada). Ionization was achieved using electrospray ionization (ESI) in the negative-ion mode with nitrogen as the nebulizer. Multiple reaction monitoring (MRM) scanning was employed for quantification. The source settings and instrument parameters for each MRM transition were optimized not only to maximize the generated deprotonated analyte molecule ([M − H]^−^) of each targeted mogroside but also to efficiently produce its characteristic fragment/product ions. The electrospray voltage was set at −4500 V, and the source temperature was 500 °C. The curtain gas (CUR), nebulizer gas (GS1), and heater gas (GS2) were set at 15, 50, and 40 psi, respectively. The compound-dependent instrumental parameters of two individual precursor-to-product ion transitions specific for each analyte, including the precursor ion, two product ions, declustering potential (DP), entrance potential (EP), collision energy (CE), and collision cell exit potential (CXP), were optimized and are listed in Table 2. The dwell time was 400 ms for each MRM transition.

**Figure A3:**
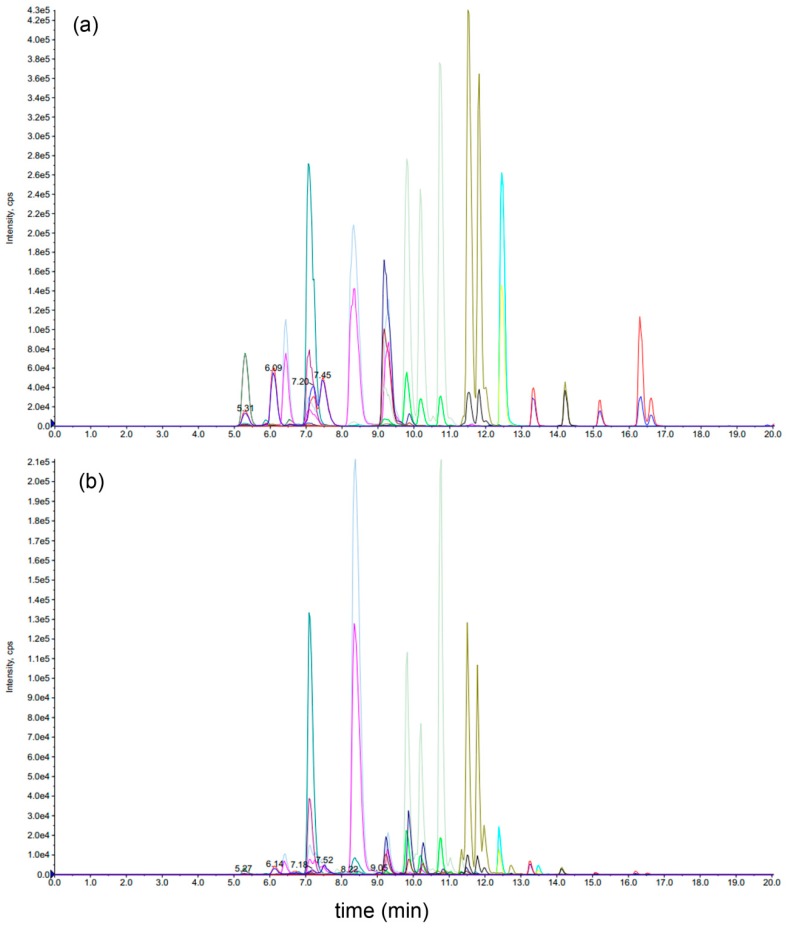
Typical LC–MS/MS total ion chromatograms. (**a**) standard solutions of 21 analytes (**b**) real sample.

LC–MS/MS chromatograms of the standards of 21 mogrosides are presented in Figure A3a, and the samples are shown in Figure A3b.

### 4.4. SNP Analysis

The dried *S. grosvenorii* materials were wiped with 75% ethanol and ground into powder. Total DNA was extracted from approximately 100 mg of the powder with the plant genomic DNA kit following the manufacturer’s instructions and dissolved in 30 μL of sterile water. *SgCS* sequences were amplified from genomic DNA by polymerase chain reaction (PCR) using the SgCS-F and SgCS-R primers (Table A4). The PCR mixture (30 µL) contained template DNA 0.8 µL, forward primer (10 µM) 0.7 µL, reverse primer (10 µM) 0.7 µL, 10× PCR Buffer 3.0 µL, KOD-Plus-Neo DNA polymerase 0.6 µL, dNTP (2 mM) 3.0 µL, MgSO_4_ (25 mM) 1.2 µL and ddH2O 20.0 µL. The PCR conditions were 94 °C for 2 min, followed by 30 cycles at 98 °C for 10 s, 59 °C for 30 s and 68 °C for 2 min 30 s, with a final incubation at 68 °C for 7 min. PCR products were examined by 1.5% agarose gel electrophoresis before bidirectional DNA sequencing on a 3730XL sequencer (Applied Biosystems, Foster City, CA, USA). Sequences were aligned using DNAman (version 8.0, Lynnon Biosoft, Quebec, Canada), and SNPs were identified by visual inspection of the alignments.

### 4.5. Cloning 4 Different Copies of SgCS Genes in Yeast

Four different copies of *SgCS* genes were codon-optimized for synthesis according to the codon bias of yeast and cloned into the *Bam*HI/*Eco*RI sites of the pCEV-G4-Km yeast expression vector under the control of the TEF1 promoter to construct pCEV-50R573L, pCEV-50C573L, pCEV-50R573Q and pCEV-50C573Q, respectively. The sequences that showed in National Center for Biotechnology Information (NCBI) database was obtained and codon-optimized as described by Qiao previously [36].

### 4.6. SgCS Mutagenesis Experiments

Mutagenesis of the 50 sites was performed using a Site-directed Mutagenesis Kit (Biomed, Beijing, China), and the corresponding degenerate primers are presented in Table A4 with the substitutions underlined. The PCR products were then purified by agarose gel electrophoresis and transformed into *Trans1-T1 E. coli*. The sequences of the mutant genes in the resulting plasmid (pCEV-G4-Km) were confirmed by Sanger sequencing using the oligonucleotide primers pCEV-Seq-F and pCEV-Seq-R (Table A4).

### 4.7. Yeast Transformation and Cell Cultivation

The plasmids were transfected into *S. cerevisiae* strain BY4742 using the Frozen-EZ yeast transformation II kit purchased from Zymo Research (CA, USA) and selected for growth on YPD plates with 200 mg/L G418. The empty pCEV-G4-Km vector was also introduced into BY4742 as a control.

The recombinant cells were first inoculated into 15 mL culture tubes containing 2 mL of YPD medium with 200 mg/L G418 and grown at 30 °C and 250 rpm to an OD600 of approximately 1.0. Flasks (250 mL) containing 100 mL of medium were then inoculated to an OD600 of 0.05 with the seed cultures. Strains were grown at 30 °C and 250 rpm for 3 days, and all optical densities at 600 nm (OD600) were measured using a Shimadzu UV-2550 spectrophotometer.

### 4.8. In Vitro Activity

The host strain *S. cerevisiae* BY4742 barboring different gene types of SgCS were grown under identical conditions to those described above and collected by centrifugation. Each yeast strain was suspended in 2 volumes of 100 mM sodium phosphate buffer, pH 7, and lysed using an Emulsiflex-C5 homogenizer. After lysis, 100 mM sodium phosphate buffer, pH 7, was added to generate a 20% slurry. A solution of 3(S)-oxidosqualene and Triton X-100 was added to the homogenate aliquots (350 µL) to a final concentration of 1 mg/mL substrate and 0.1% Triton X-100. After 0.5, 1, 3, 5, and 10 h, the reactions were terminated by adding two volumes of ethanol. The denatured protein was removed by centrifugation, and the supernatant was concentrated to dryness under a nitrogen stream. The residue was resuspended in n-hexane and analyzed by GC-MS.

### 4.9. GC-MS Analysis of Yeast and Plant Extracts

Cells were collected by centrifugation at 10,000× *g* for 5 min, refluxed with 5 mL of 20% KOH/50% ethanol and extracted three times with the same volume of n-hexane. The combined extracts were dried under reduced pressure distillation and dissolved in 1 mL of n-hexane.

GC-MS was performed on a Thermo Scientific ISQ Single Quadrupole GC-MS (Thermo Scientific, Waltham, MA USA). Separation of the components of the nonpolar active ingredients was carried out on an HP-5 MS capillary column (5% phenyl and 95% dimethylpolysiloxane, 30 m × 0.25 mm × 0.25 μm). An electron ionization system with an ionization energy of 70 eV was used in the mass spectrometer. Helium gas was used as the carrier gas, and the carrier flow rate was 1.5 mL/min. The temperatures of both the injector and MS transfer line were 250 °C, and the ion source temperature was 220 °C. The initial oven temperature was 70 °C, which was held for 2 min, and the temperature was then programmed to linearly increase at a rate of 20 °C/min up to 260 °C and finally increase by 10 °C/min up to 300 °C, where it was held for 10 min. A 1 μL sample was injected automatically into the monitoring system in 1:10 split mode. Under these conditions, squalene and cucurbitadienol eluted at 14.73 and 18.85 min, respectively.

### 4.10. Data Processing and Statistics

Data were expressed as the mean ± standard deviation. IBM SPSS statistics software 22.0 (SPSS Inc., Chicago, IL, USA) was used for data analysis, including descriptive statistics of the data, correlation analysis of the factors, regression analysis and the chi-square test. The boxplots were generated with Origin 8 (OriginLab Co., Northampton, MA, USA).

## 5. Conclusions

In this study, we conducted a biosynthesis-based secondary metabolomics analysis of 15 *S. grosvenorii* varieties. A high catalytic efficiency SgCS enzyme was obtained by SNP analysis and site-directed mutation. The genotypes and chemical differences of 15 *S. grosvenorii* varieties were revealed by SNP analysis of cucurbitadienol and quantitative analysis of secondary metabolomics, respectively. The 15 varieties showed significant differences in their secondary metabolite profiles. A total of four wild-type SgCS protein variants based on two missense mutation SNP sites were discovered. Moreover, a site-directed mutant, namely, 50K573L, produced a 33% enhancement in efficiency compared to wild-type 50R573L. Our findings thus identify a novel cucurbitadienol synthase allele correlates with high efficiency and provide new insight into molecular breeding of *S. grosvenorii*. Future studies are planned to include more varieties and functional genes, such as CYP450 and UGT, to produce high-quality *S. grosvenorii*. Gene editing technology can be used to knock out the “bad gene” and transfer the “better gene” into the elite *S. grosvenori*i germ. The “high catalytic efficiency” plant can be obtained and verified by PCR, Western Blot analysis and so on, which will lay the foundation for molecular design breeding.

## Figures and Tables

**Table 1:** Determination of kinetic parameters of four wild types of SgCS (50R573L, 50C573L, 50R573Q, and 50C573Q). The kinetic parameters of the reaction were assayed with 3(S)-oxidosqualene as sbustrate at 30 °C and pH 7.0.

	Km (µM)	Kcat (min^−1^)	Kcat/Km (µM^−1^ min^−1^)	Specific Activity (nmol min^−1^ mg^−1^)	Relative Activity (%)
50R573L	0.29	0.88	2.98	10.24	100
50C573L	0.32	0.77	2.41	8.28	80.8
50R573Q	ND ^(a)^	ND	ND	ND	-
50C573Q	ND	ND	ND	ND	-

^(^^a)^: not detected.

**Table 2:** MS parameters of 21 mogrosides.

	Analytes	Molecular Formula	t_R_ (min)	*m*/*z* Precursor	*m*/*z* Productions	DP (V)	CE (eV)
M2	MIIA	C_42_H_72_O_14_	16.4	799.5	637.6 ^(a)^/475.5	−170	−65
	MIIA1	C_42_H_72_O_14_	16.7	799.5	637.6 ^(a)^/475.5	−170	−65
	MIIA2	C_42_H_72_O_14_	15.27	799.5	637.6 ^(a)^/475.5	−170	−65
	MIIE	C_42_H_72_O_14_	13.4	799.5	637.6 ^(a)^/475.5	−170	−65
M3	MIII	C_48_H_82_O_19_	11.56	961.6	799.4 ^(a)^/637.3	−170	−70
	MIIIE	C_48_H_82_O_19_	11.85	961.6	799.4 ^(a)^/637.3	−170	−70
	MIIIA1	C_48_H_82_O_19_	14.31	961.6	799.4 ^(a)^/637.3	−170	−70
	MIIIA2	C_48_H_82_O_19_	12.07	961.6	799.4 ^(a)^/637.3	−170	−70
M4	11-O-SI	C_54_H_90_O_24_	9.32	1121.6	959.6 ^(a)^/797.4	−220	−75
	SI	C_54_H_92_O_24_	9.82	1123.6	961.6 ^(a)^/799.2	−220	−75
	MIVA	C_54_H_92_O_24_	10.20	1123.6	961.6 ^(a)^/799.2	−220	−75
	MIVE	C_54_H_92_O_24_	10.77	1123.6	961.6 ^(a)^/799.2	−220	−75
M5	11-D-O-MV	C_60_H_102_O_28_	12.48	1269.8	1107.7 ^(a)^/945.6	−230	−90
	11-E-MV	C_60_H_100_O_29_	7.12	1283.8	1121.6 ^(a)^/959.5	−220	−85
	MV	C_60_H_102_O_29_	8.44	1285.8	1123.7 ^(a)^/961.7	−220	−90
	O-MV	C_60_H_102_O_29_	6.52	1285.8	1123.7 ^(a)^/961.7	−220	−90
	IMV	C_60_H_102_O_29_	9.35	1285.8	1123.7 ^(a)^/961.7	−220	−90
M6	11-O-MVI	C_66_H_110_O_34_	5.29	1445.8	1283.5 ^(a)^/1121.7	−240	−100
	MVI	C_66_H_112_O_34_	6.15	1447.8	1285.6 ^(a)^/1123.6	−230	−105
	MVIA	C_66_H_112_O_34_	7.33	1447.8	1285.6 ^(a)^/1123.6	−230	−105
	MVIB	C_66_H_112_O_34_	7.13	1447.8	1285.6 ^(a)^/1123.6	−230	−105
	Source temperature (°C)			500			
	Ionization voltage (V)			−4500			
	Ion source (GS1) setting (psi)			50			
	Ion source (GS2) setting (psi)			40			
	Curtain gas setting (psi)			15			
	CAD			high			
	Dwell time (ms)			400			
	EP (V)			−10			
	CXP (V)			−15			

^(a)^ Product ion used for quantification. Abbreviations: t_R_, retention time. DP, declustering potential; CE, collision energy; CAD, Collision activation dissociation; EP, Entrance potential; CXP, Collision cell exit potential.

## Abbreviations

11-D-O-MV	11-deoxymogroside V
11-E-MV	11-epi-mogroside V
O-MV	11-oxo-mogroside V
11-O-SI	11-O-siamenoside I
11-O-MVI	11-oxo-mogroside VI
CS	cucurbitadienol synthase
CYP450	cytochrome P450s
EPH	epoxide hydrolases
IMV	isomogroside V
MIIA	mogroside IIA
MIIA1	mogroside IIA1
MIIA2	mogroside IIA2
MIIE	mogroside IIE
MIII	mogroside III
MIIIA1	mogroside IIIA1
MIIIA2	mogroside IIIA2
MIIIE	mogroside IIIE
MIVE	mogroside IVE
MIVA	mogroside IVA
MV	mogroside V
MVI	mogroside VI
MVIA	mogroside VI A
MVIB	mogroside VIB
SI	siamenoside I
SNP	single nucleotide polymorphism
SQE	squalene epoxidases
UGT	UDP-glucosyltransferase

## Appendix A

Figure A1. Hierarchical clustering analysis of S. grosvenorii varieties.

Figure A2. Alignment of PCR fragment showing the SNP sites of SgCS.

Figure A3. Typical LC–MS/MS total ion chromatograms.

Table A1. Chemical structures of 21 mogrosides from luohanguo.

Table A2. Contents (mg/g) of 21 mogrosides in tested samples.

Table A3. Contents (mg/g) of squalene and cucurbitadienol in tested samples.

Table A4. Primers used in this study.

**Table A1:** Chemical structures of 21 mogrosides from luohanguo.

Commom Name	CAS No.	-R_1_	-R_2_	-R_3_
mogroside IIA	1613527-65-3	-H		
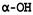
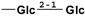
mogroside IIA1	88901-44-4	-H		
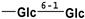
mogroside IIA2	88901-45-5			-H
mogroside IIe	88901-38-6	^a^		
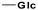
mogroside III	130567-83-8			
mogroside IIIe	88901-37-5			
mogroside IIIA1	88901-42-2	-H		
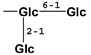
mogroside IIIA2	88901-43-3			
11-O-siamenoside I	1793003-50-5			
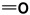
mogroside IVa	88901-41-1			
siamenoside I	126105-12-2			
mogroside IVE	89590-95-4			
11-deoxymogroside V	1707161-17-8		-H	
11-epi-mogroside V	2146088-12-0			
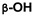
mogroside V	88901-36-4			
11-oxo-mogroside V	126105-11-1			
isomogroside V	1126032-65-2			
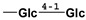
11-oxo-mogroside VI	1421942-59-7			
mogroside VI	89590-98-7			
mogroside VI A	2146088-13-1			
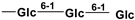
mogroside VI B	2149606-17-5			
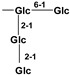

^a^ Glc = *β-D-glucopyranosyl.*

**Table A2:** Contents (mg/g) of 21 mogrosides in tested samples (*n* = 3, mean ± SD).

	MVI	MVIA	MVIB	11-O-MVI	11-O-MV	MV	IMV	11-E-MV	11-D-O-MV	SI	MIVA	MIVE	11-O-SI	MIII	MIIIE	MIIIA2	MIIIA1	MIIE	MIIA2	MIIA	MIIA1
**E1**	0.66 ± 0.19	0.34 ± 0.10	0.89 ± 0.26	0.23 ± 0.07	0.33 ± 0.07	12.31 ± 2.83	1.75 ± 0.42	3.87 ± 1.01	0.26 ± 0.08	1.16 ± 0.26	0.74 ± 0.16	1.31 ± 0.30	0.30 ± 0.07	0.04 ± 0.01	0.68 ± 0.12	0.04 ± 0.01	0.29 ± 0.07	ND	0.14 ± 0.04	0.19 ± 0.05	ND
**E2**	0.35 ± 0.02	0.15 ± 0.02	0.40 ± 0.02	0.11 ± 0.01	0.27 ± 0.00	10.43 ± 0.35	1.05 ± 0.07	2.91 ± 0.09	0.29 ± 0.02	0.83 ± 0.05	1.80 ± 0.05	1.02 ± 0.03	0.18 ± 0.01	0.61 ± 0.03	0.33 ± 0.03	0.19 ± 0.02	0.12 ± 0.00	0.09 ± 0.01	0.06 ± 0.01	0.04 ± 0.00	ND
**E3**	0.71 ± 0.08	0.30 ± 0.02	0.84 ± 0.03	0.22 ± 0.01	0.34 ± 0.01	10.90 ± 0.48	0.93 ± 0.04	4.09 ± 0.13	0.45 ± 0.03	1.12 ± 0.05	0.88 ± 0.04	1.45 ± 0.10	0.33 ± 0.02	0.13 ± 0.01	0.89 ± 0.05	0.04 ± 0.01	0.23 ± 0.01	0.01 ± 0.00	0.12 ± 0.02	0.14 ± 0.01	ND
**E5**	0.74 ± 0.06	0.36 ± 0.03	0.89 ± 0.07	0.19 ± 0.01	0.39 ± 0.03	12.68 ± 0.35	1.41 ± 0.04	3.80 ± 0.28	0.50 ± 0.05	0.50 ± 0.04	1.74 ± 0.12	0.08 ± 0.01	0.04 ± 0.00	ND	0.02 ± 0.00	0.09 ± 0.01	ND	0.08 ± 0.01	ND	ND	ND
**E12**	0.56 ± 0.04	0.29 ± 0.01	0.67 ± 0.04	0.14 ± 0.00	0.28 ± 0.03	10.80 ± 0.43	0.89 ± 0.05	2.91 ± 0.20	0.34 ± 0.03	1.03 ± 0.09	1.72 ± 0.14	1.08 ± 0.06	0.21 ± 0.01	0.39 ± 0.03	0.49 ± 0.05	0.12 ± 0.01	0.25 ± 0.03	0.02 ± 0.00	0.08 ± 0.01	0.09 ± 0.00	ND
**E23**	0.44 ± 0.02	0.21 ± 0.02	0.59 ± 0.02	0.12 ± 0.00	0.28 ± 0.01	12.15 ± 0.06	1.28 ± 0.05	2.82 ± 0.07	0.44 ± 0.01	1.07 ± 0.03	0.81 ± 0.03	0.91 ± 0.01	0.17 ± 0.01	0.07 ± 0.00	0.80 ± 0.02	0.03 ± 0.00	0.17 ± 0.01	ND	0.06 ± 0.01	0.05 ± 0.00	ND
**E29**	0.46 ± 0.04	0.18 ± 0.00	0.45 ± 0.05	0.11 ± 0.01	0.23 ± 0.02	10.76 ± 0.36	0.96 ± 0.08	2.54 ± 0.14	0.29 ± 0.02	0.74 ± 0.05	0.37 ± 0.03	0.99 ± 0.03	0.14 ± 0.00	0.01 ± 0.00	0.26 ± 0.01	0.03 ± 0.00	0.12 ± 0.01	ND	0.06 ± 0.01	0.05 ± 0.01	ND
**S2**	0.69 ± 0.07	0.30 ± 0.02	0.76 ± 0.05	0.18 ± 0.01	0.41 ± 0.02	13.49 ± 0.63	0.75 ± 0.01	3.91 ± 0.18	0.54 ± 0.05	1.21 ± 0.09	0.85 ± 0.06	1.33 ± 0.10	0.21 ± 0.01	0.05 ± 0.00	0.47 ± 0.04	0.05 ± 0.01	0.27 ± 0.02	ND	0.12 ± 0.01	0.09 ± 0.01	ND
**S3**	0.24 ± 0.01	0.14 ± 0.01	0.34 ± 0.02	0.05 ± 0.00	0.22 ± 0.02	10.84 ± 0.45	0.61 ± 0.02	2.02 ± 0.13	0.25 ± 0.01	0.72 ± 0.04	0.46 ± 0.02	0.75 ± 0.04	0.09 ± 0.01	0.01 ± 0.00	0.08 ± 0.00	ND	0.15 ± 0.01	ND	0.07 ± 0.01	0.04 ± 0.00	ND
**S10**	0.13 ± 0.00	0.04 ± 0.01	0.17 ± 0.01	0.03 ± 0.00	0.05 ± 0.00	4.86 ± 0.05	0.35 ± 0.02	1.01 ± 0.01	0.01 ± 0.00	0.26 ± 0.00	0.16 ± 0.00	0.44 ± 0.02	0.07 ± 0.00	ND	0.13 ± 0.00	ND	0.05 ± 0.00	ND	0.02 ± 0.01	0.04 ± 0.00	ND
**S13**	0.27 ± 0.01	0.10 ± 0.01	0.17 ± 0.01	0.05 ± 0.00	0.08 ± 0.01	7.24 ± 0.20	0.28 ± 0.01	1.10 ± 0.02	0.28 ± 0.00	0.82 ± 0.02	0.18 ± 0.00	2.10 ± 0.01	0.13 ± 0.00	0.01 ± 0.00	0.88 ± 0.02	0.07 ± 0.00	0.08 ± 0.00	ND	0.02 ± 0.00	0.11 ± 0.00	ND
**C2**	0.35 ± 0.03	0.16 ± 0.02	0.47 ± 0.04	0.10 ± 0.01	0.17 ± 0.02	9.91 ± 0.61	1.14 ± 0.10	1.83 ± 0.17	0.25 ± 0.03	0.76 ± 0.07	0.23 ± 0.03	1.10 ± 0.10	0.18 ± 0.01	0.11 ± 0.01	1.90 ± 0.15	0.08 ± 0.01	0.10 ± 0.01	ND	0.02 ± 0.00	0.99 ± 0.09	0.05 ± 0.01
**C3**	0.37 ± 0.03	0.16 ± 0.02	0.41 ± 0.05	0.14 ± 0.01	0.23 ± 0.05	10.70 ± 0.46	0.87 ± 0.26	2.87 ± 0.60	0.29 ± 0.01	1.01 ± 0.14	0.22 ± 0.04	1.35 ± 0.11	0.25 ± 0.03	0.04 ± 0.05	0.97 ± 0.81	0.02 ± 0.00	0.10 ± 0.02	ND	ND	0.38 ± 0.48	0.02 ± 0.05
**C6**	0.37 ± 0.02	0.16 ± 0.01	0.46 ± 0.02	0.11 ± 0.01	0.23 ± 0.01	10.20 ± 0.34	0.89 ± 0.04	2.40 ± 0.09	0.26 ± 0.02	1.06 ± 0.04	0.44 ± 0.02	1.88 ± 0.06	0.27 ± 0.01	0.04 ± 0.00	0.79 ± 0.03	0.10 ± 0.01	0.14 ± 0.01	ND	0.07 ± 0.01	0.13 ± 0.01	ND
**W4**	0.31 ± 0.03	0.22 ± 0.02	0.40 ± 0.03	0.07 ± 0.01	0.22 ± 0.02	10.10 ± 0.56	0.37 ± 0.04	2.05 ± 0.13	0.21 ± 0.02	0.73 ± 0.04	0.33 ± 0.03	0.98 ± 0.04	0.14 ± 0.01	0.02 ± 0.00	0.55 ± 0.02	0.03 ± 0.01	0.15 ± 0.01	ND	0.04 ± 0	0.25 ± 0.01	ND
**Average Content**	0.44	0.21	0.53	0.12	0.25	10.49	0.90	2.67	0.31	0.87	0.73	1.12	0.18	0.10	0.62	0.06	0.15	0.01	0.06	0.17	0.00
**Coefficient of Variation (%)**	43.37	46.02	45.88	50.25	40.14	21.12	45.94	37.29	41.87	30.98	78.03	44.31	46.9	166.85	82.1	80.17	58.66	--	88.59	157.39	--

**Table A3:** Contents (mg/g) of squalene and cucurbitadienol in tested samples (*n* = 3, mean ± SD).

	E1	E2	E3	E5	E12	E23	E29	S2	S3	S10	S13	C2	C3	C6	W4	Average Content	CV (%)
**Squalene**	0.10 ± 0.01	0.28 ± 0.13	0.03 ± 0.00	0.08 ± 0.01	0.07 ± 0.01	0.07 ± 0.01	0.24 ± 0.09	0.10 ± 0.02	0.06 ± 0.02	0.07 ± 0.00	0.17 ± 0.04	1.24 ± 0.15	0.39 ± 0.13	0.17 ± 0.01	0.21 ± 0.05	0.22	135.03
**Cucurbitadienol**	0.48 ± 0.05	0.62 ± 0.24	0.23 ± 0.05	0.25 ± 0.02	0.22 ± 0.00	0.20 ± 0.01	0.36 ± 0.07	0.27 ± 0.06	0.18 ± 0.04	0.17 ± 0.02	0.56 ± 0.07	1.80 ± 0.19	1.01 ± 0.22	0.57 ± 0.03	0.55 ± 0.11	0.50	86.03

**Table A4:** Primers used in this study.

SgCS-F	ATGTGGAGGTTAAAGGTC
SgCS-R	TTATTCAGTCAAAACCCG
pCEV- Seq-F	CGATGACCTCCCATTGATA
pCEV- Seq-R	CGTTCTTAATACTAACATAACT
50A-F	ATTGTTGCAAGTTCATAAAGCT**GCT**AAAGCTTTTCATGATGATAGA
50A-R	TCTATCATCATGAAAAGCTTT**AGC**AGCTTTATGAACTTGCAACAAT
50C-F	ATTGTTGCAAGTTCATAAAGCT**TGT**AAAGCTTTTCATGATGATAGA
50C-R	TCTATCATCATGAAAAGCTTT**ACA**AGCTTTATGAACTTGCAACAAT
50D-F	ATTGTTGCAAGTTCATAAAGCT**GAT**AAAGCTTTTCATGATGATAGA
50D-R	TCTATCATCATGAAAAGCTTT**ATC**AGCTTTATGAACTTGCAACAAT
50E-F	ATTGTTGCAAGTTCATAAAGCT**GAA**AAAGCTTTTCATGATGATAGA
50E-R	TCTATCATCATGAAAAGCTTT**TTC**AGCTTTATGAACTTGCAACAAT
50H-F	ATTGTTGCAAGTTCATAAAGCT**CAT**AAAGCTTTTCATGATGATAGA
50H-R	TCTATCATCATGAAAAGCTTT**ATG**AGCTTTATGAACTTGCAACAAT
50K-F	ATTGTTGCAAGTTCATAAAGCT**AAA**AAAGCTTTTCATGATGATAGA
50K-R	TCTATCATCATGAAAAGCTTT**TTT**AGCTTTATGAACTTGCAACAAT

The nucleotides in bold and underlined were the corresponding mutant sites.
